# Supplementary material for: Optimizing fungal DNA extraction and purification for Oxford Nanopore untargeted shotgun metagenomic sequencing from simulated hemoculture specimens
Source: mSystems. 2025 Apr 8;10(6):e01166-24. doi: 10.1128/msystems.01166-24 (PMC12172461; doi:10.1128/msystems.01166-24)
Supplement: Table S1 — The metadata of the six studied fungal isolates. [file msystems.01166-24-s0001.docx]

**Table S1**: The metadata of the six studied fungal isolates

| **Strain** | **Species** | **BioSample accession** | **Isolated from** | **Method of identification** |
| --- | --- | --- | --- | --- |
| CUAFU24 | *Aspergillus fumigatus* | SAMN39985754 | Bronchoalveolar lavage | LCB/PCR |
| CUCNE41 | *Cryptococcus neoformans* | SAMN39985753 | Hemoculture | Vitek II™/PCR |
| CUCGL01 | *Nakaseomyces glabratus* | SAMN39985752 | Sputum | Vitek II™/PCR |
| CUPIN58 | *Pythium insidiosum* | SAMN39985757 | Vascular tissue | PCR |
| CURHI38 | *Rhizopus microsporus* | SAMN39985756 | Bronchoalveolar lavage | LCB/PCR |
| CUSSC26 | *Sporothrix schenckii* | SAMN39985755 | Tissue | LCB/PCR |

Notes: LCB: Lactophenol Cotton Blue wet mount, PCR: Polymerase Chain Reaction, Vitek II™: Biochemical test (bioMérieux, France)
