# Supplementary material for: Optimizing fungal DNA extraction and purification for Oxford Nanopore untargeted shotgun metagenomic sequencing from simulated hemoculture specimens
Source: mSystems. 2025 Apr 8;10(6):e01166-24. doi: 10.1128/msystems.01166-24 (PMC12172461; doi:10.1128/msystems.01166-24)
Supplement: Table S2 — Comparison of the extraction steps of the newly designed DNA extraction protocol compare with the conventional DNA extraction protocol for fungi. [file msystems.01166-24-s0002.docx]

| New DNA Extraction Protocol | | Conventional DNA Extraction Protocol | |
| --- | --- | --- | --- |
| Step | Duration | Step | Duration |
| Human DNA depletion protocol | 30 minutes | Lysis buffer incubation  (0.2 M NaCl, 0.02 M EDTA, 0.04 M Tris-HCl, 10% w/v SDS) | 3 hours |
| Vortexing with beads  (optional for filamentous molds) | 20 minutes | Bead beating | 5 minutes |
| Lysis buffer incubation  (10% SDS, 0.04 M Tris-HCl, 0.02 M EDTA, 100 μg/mL proteinase K, 2% CTAB, 1% PVP, and 3 M NaCl) | 3 hours | DNA isolation  (Phenol chloroform DNA isolation) | 4 to more than 12 hours |
| Ultracentrifugation | 15 minutes |  |  |
| DNA isolation (paramagnetic beads: Automation) | 45 minutes |  |  |
| Total | Approximately 5 hours |  | Approximately 7 to more than 15 hours |

**Table S2:** Comparison of the extraction steps of the newly designed DNA extraction protocol compare with the conventional DNA extraction protocol for fungi.
